# Supplementary material for: Mirage2’s high-quality spliced protein-to-genome mappings produce accurate multiple-sequence alignments of isoforms
Source: PLoS One. 2023 May 8;18(5):e0285225. doi: 10.1371/journal.pone.0285225 (PMC10166558; doi:10.1371/journal.pone.0285225)
Supplement: Attachment — Submitted filename: PLOSOne-Mirage2-revision-coverletter.pdf [file pone.0285225.s002.pdf]

UNIVERSITY OF MONTANA

Feb 26, 2023

To Whom It May Concern:

We thank the reviewers for their thoughtful feedback on our manuscript, "*Mirage2*'s high-quality spliced protein-to-genome mappings produce accurate multiple-sequence alignments of isoforms". Following the recommendations provided by the reviewers and editor, we have made the following improvements to the manuscript (highlighted in red within the submitted revision):

1. We have expanded the description of the relevant background and state of the field in the Introduction section.
2. Our description of the function of *BLAT*, both generally and within the context of our decision to use it within *Mirage2*, is much more robust in the Methods section.
3. In addition to the benefits described in the Discussion section, we have added a quantitative comparison of the performance benefits of *Mirage2* over the original *Mirage* implementation in the Results section, emphasizing that *Mirage2* halves the number of unmapped sequences from the original tool and that we have significantly improved the number of sequences mapped using our preferred method (*FastMap*).
4. We have added information about the impact of deactivating *BLAT* on MSA quality to the Results section.
5. Minor updates have been made to several of the figures.
6. We have improved Figure 2 to indicate that it is complete as-is. Because of the importance of this novel file format to the use of *Mirage2*, we believe that it is valuable for Figure 2 to remain in the manuscript.
7. Funding information has been removed from the Acknowledgements section. We would like to ensure that the submitted Funding Statement now reads:
   "A.J.N. and T.J.W acknowledge funding from the National Institute of General Medical Sciences (NIGMS, https://www.nigms.nih.gov/), National Institutes of Health (NIH), grants GM123487 and GM132600, and from the National Human Genome Research Institute (NHGRI, NIH, https://www.genome.gov/), grant HG012283. The funders had no role in study design, data collection and analysis, decision to publish, or preparation of the manuscript."
8. We have adjusted the bibliography to meet Vancouver-style formatting guidelines.
9. We have made a minimal underlying dataset available at the following stable URL:
   https://osf.io/7pgav/ (DOI: 10.17605/OSF.IO/7PGAV)

We hope that these are all welcome changes and that the improved manuscript satisfies the quality and formatting guidelines for publishing with *PLOSOne*.

Regards,

Alexander Nord
Division of Biological Sciences
University of Montana – Missoula
